# Echolocating Bats Use a Nearly Time-Optimal Strategy to Intercept Prey

**DOI:** 10.1371/journal.pbio.0040108

**Published:** 2006-04-18

**Authors:** Kaushik Ghose, Timothy K Horiuchi, P. S Krishnaprasad, Cynthia F Moss

**Affiliations:** **1**Neuroscience and Cognitive Science Program, University of Maryland, College Park, Maryland, United States of America; **2**Department of Psychology, University of Maryland, College Park, Maryland, United States of America; **3**Department of Electrical and Computer Engineering, University of Maryland, College Park, Maryland, United States of America; **4**Institute for Systems Research, University of Maryland, College Park, Maryland, United States of America; California Institute of TechnologyUnited States of America

## Abstract

Acquisition of food in many animal species depends on the pursuit and capture of moving prey. Among modern humans, the pursuit and interception of moving targets plays a central role in a variety of sports, such as tennis, football, Frisbee, and baseball. Studies of target pursuit in animals, ranging from dragonflies to fish and dogs to humans, have suggested that they all use a *constant bearing* (CB) strategy to pursue prey or other moving targets. CB is best known as the interception strategy employed by baseball outfielders to catch ballistic fly balls. CB is a time-optimal solution to catch targets moving along a straight line, or in a predictable fashion—such as a ballistic baseball, or a piece of food sinking in water. Many animals, however, have to capture prey that may make evasive and unpredictable maneuvers. Is CB an optimum solution to pursuing erratically moving targets? Do animals faced with such erratic prey also use CB? In this paper, we address these questions by studying prey capture in an insectivorous echolocating bat. Echolocating bats rely on sonar to pursue and capture flying insects. The bat's prey may emerge from foliage for a brief time, fly in erratic three-dimensional paths before returning to cover. Bats typically take less than one second to detect, localize and capture such insects. We used high speed stereo infra-red videography to study the three dimensional flight paths of the big brown bat, *Eptesicus fuscus,* as it chased erratically moving insects in a dark laboratory flight room. We quantified the bat's complex pursuit trajectories using a simple delay differential equation. Our analysis of the pursuit trajectories suggests that bats use a *constant absolute target direction* strategy during pursuit. We show mathematically that, unlike CB, this approach minimizes the time it takes for a pursuer to intercept an unpredictably moving target. Interestingly, the bat's behavior is similar to the interception strategy implemented in some guided missiles. We suggest that the time-optimal strategy adopted by the bat is in response to the evolutionary pressures of having to capture erratic and fast moving insects.

## Abstract 2

Analysis of the three dimensional flight paths of the big brown bat reveals a similar strategy to intercept targets as used by some guided missiles.

## Introduction

Echolocating bats forage on the wing in darkness. Their primary sensory system for hunting in the dark is echolocation [ 1, 2]. They emit short pulses of broadband sound, predominantly at ultrasonic frequencies, to derive information from the returning echoes. Bats engage in a natural version of the “homicidal chauffeur” game [ 3], preying upon small, fast, erratically moving insects that may fly in the open only for brief periods at a time [ 4– 6]. A bat therefore has a fleeting time window within which to detect, localize, and capture its prey. A complete insect chase from detection to capture typically takes less than one second [ 7]. The short time window available for capturing such highly maneuverable and unpredictable prey would suggest evolutionary pressure for the bat to adopt a pursuit strategy appropriate for its needs. Using high-speed video and audio recordings of the big brown bat *(Eptesicus fuscus)* chasing tethered and free-flying insects in a laboratory flight-room, we show that the echolocating bat uses a previously undescribed pursuit strategy while capturing prey. We argue in this paper that this strategy minimizes time-to-capture of an unpredictably moving insect.

Previous studies of fish [ 8], dragonflies [ 9], and humans [ 10– 12] show that a wide variety of animals use a constant bearing (CB) strategy during pursuit. Here, the animal keeps the angle between its heading (velocity vector) and the target a constant as it closes the target range. Additionally, the animal attempts to move in a straight line—a condition that prevents spiral paths about a target [ 12]. This strategy, as a means to detect a collision course with another object, has been known anecdotally for hundreds of years to sailors and more recently to airplane pilots and car drivers and is known as “constant bearing, decreasing range.” It was formalized in the 1960s in the human psychology literature [ 13, 14]. This research has led to the hypothesis that the CB strategy is widespread because it involves the use of a perceptual invariant—by simply nulling the rate of change in the visual angle to a target, animals can pursue a moving object [ 15].

**Figure 1:**
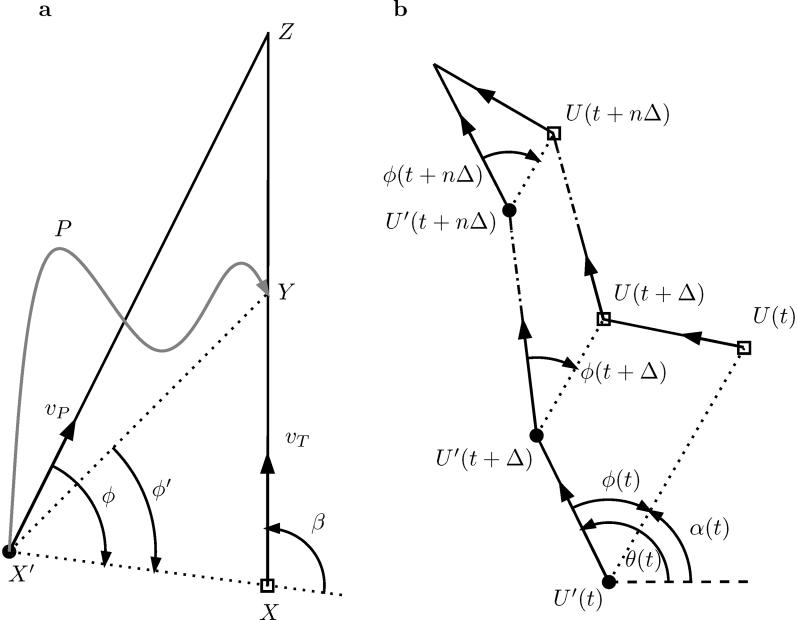
Time-Optimal Strategies to Intercept a Target (A) The target (square), which starts at position *X,* moves in a straight line at a constant speed *v _T_*. The pursuer (solid disk), which starts at position *X′,* moves at a constant speed *v _p_*. The straight-line intercept *X′Z,* where *φ* is given by Equation 1, is the shortest intercept path possible. Quicker intercepts such as *X′Y* are not possible (see text). (B) The target (square), which starts at position *U*( *t*), moves erratically, changing both speed and direction. The pursuer (solid disk) starts at position *U′*( *t*) *.* The erratic target motion can be approximated by infinitesimal constant velocity segments (such as *U*( *t*) *U*( *t+*Δ) where Δ → 0). There is no globally minimum-time intercept for truly erratic targets. A pursuer can follow a locally time-optimum path by adjusting its motion such that *φ* for each infinitesimal segment is given by Equation 1. In such a condition the bearing lines drawn from pursuer to target ( *U′*( *t*) *U*( *t*), etc.) remain parallel to each other (α has a fixed value) while the target bearing ( *φ*) and pursuer heading direction ( *θ*) may change continually. *α* and *θ* are measured with respect to an external, fixed reference frame.



The CB strategy has been successful in explaining pursuit behavior when the target moves at constant velocity and the pursuer moves at constant speed. Under the condition of constant target velocity a pursuer following a CB strategy intercepts the target by moving along a straight line while holding a fixed target bearing (see Figure 1A) given by

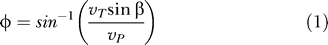



If a pursuer is too slow ( *v _p_ < v _T_* sin β), it cannot intercept the target, and there is no solution to Equation 1. If *v _p_ > v _T_* sin β, then there are two solutions to Equation 1, only one of which causes the distance between the pursuer and the target to decrease.

Under the condition of constant target velocity, when a pursuer follows a CB strategy, it intercepts the target in minimum time. We offer a proof of this by contradiction: when holding a CB, the pursuer follows a straight path *X′Z* to intercept the target in time *T* ( Figure 1A). Suppose there is another path *X′PY* (not necessarily a straight line) that would allow the pursuer to intercept the target in shorter time *T′,* at position *Y*. In that case



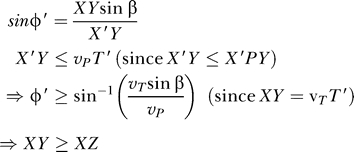



a contradiction, implying *X′Z* is the shortest interception path available to the pursuer. This demonstrates that *φ,* defined by Equation 1, is the optimum bearing that leads to interception in minimum time. Hence we will refer to this value as *φ _opt_* in what follows.

Bats often pursue targets that move unpredictably. The path of such a target may be broken into infinitesimally short linear segments each of constant velocity ( Figure 1B). If the pursuer follows an optimum-bearing intercept path for each linear segment, then it minimizes time-to-intercept locally, for the duration of that segment. In general the optimum bearing *φ _opt_* will vary from segment to segment. If the linear segments are long enough then the animal could still use the CB strategy to converge to the optimum bearing (given by Equation 1) for each segment. The pursuit will then consist of relatively long periods of CB, interspersed with short periods when the target adopts a new velocity and the pursuer converges to a new CB. A study on dogs catching Frisbees supports this idea [ 16]. If the target motion is sufficiently erratic, however, an animal attempting to execute the CB strategy will never converge to the optimum bearing for any segment.

In the case of an erratically moving target, a pursuer can maintain an optimum bearing using a different strategy. The velocities of the target and pursuer may be decomposed into two components, one parallel to the line joining them (e.g., along *U′U,* Figure 1B) and one perpendicular to this line (transverse component). When an animal maintains optimum bearing, the transverse component of the velocities of the pursuer and target are matched. This means that the absolute direction to the target (the direction of the line *U′U,* also described by the angle *α*) remains constant. If the pursuer follows a constant absolute target direction (CATD) strategy where it maneuvers to minimize changes in the absolute direction to the target, the pursuer can maintain the optimum bearing for each instant of the pursuit. The pursuer can follow this strategy by adjusting both its direction of motion and its speed, ensuring *v _p_ > v _T_* sin β as mentioned previously.

In this study we investigated whether the pursuit of erratically moving insects by E. fuscus is best described as CB (as reported in many other animals) or whether the bat uses a CATD strategy to meet its behavioral requirements. Our results indicate that E. fuscus follows a CATD strategy.

## Results

We trained eight bats to fly in a large, dark, instrumented flight room and capture both tethered and free-flying insect prey. The bat and insect prey were recorded using two high speed infrared video cameras. The flight paths of the bat and its prey were reconstructed from the stereo video frames. Simultaneously, a custom built, U-shaped array of 16 microphones recorded horizontal cross-sections of the sonar beam pattern emitted by the bats. E. fuscus emits echolocation cries through the open mouth, so the axis of the sonar beam is aligned with the axis of the head. These measurements, therefore, allowed us to compute the horizontal direction of the bat's head as it chased its prey [ 17]. The bat was allowed to fly in the room for a random period of time (10–30 s), after which the insect prey was released into the room. Each bat was tested individually as it chased a single prey item presented in the room. A trial consisted of the release of the insect and the first attempt by the bat to capture it.

We define for every instant *t,*



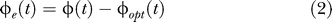



the difference between the actual bearing to the target, *φ*( *t*) *,* and the optimum bearing, *φ _opt_*( *t*) *,* given by Equation 1.

If the bat were maneuvering to follow the optimum bearing perfectly, *φ _e_* should decrease to zero during insect pursuit. If the bat's behavior is better explained by a CATD strategy than a CB strategy, then the rate of change of the absolute target direction should be zero ( 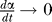). From Figure 1B we see that *α = θ + φ* (for two-dimensional angles, and for azimuth and elevation components of three-dimensional angles). So



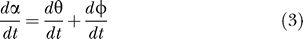



and for 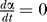, we have



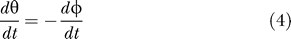



On the other hand, if the bat were following a CB strategy, *φ* should remain a constant ( 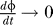).

**Figure 2:**
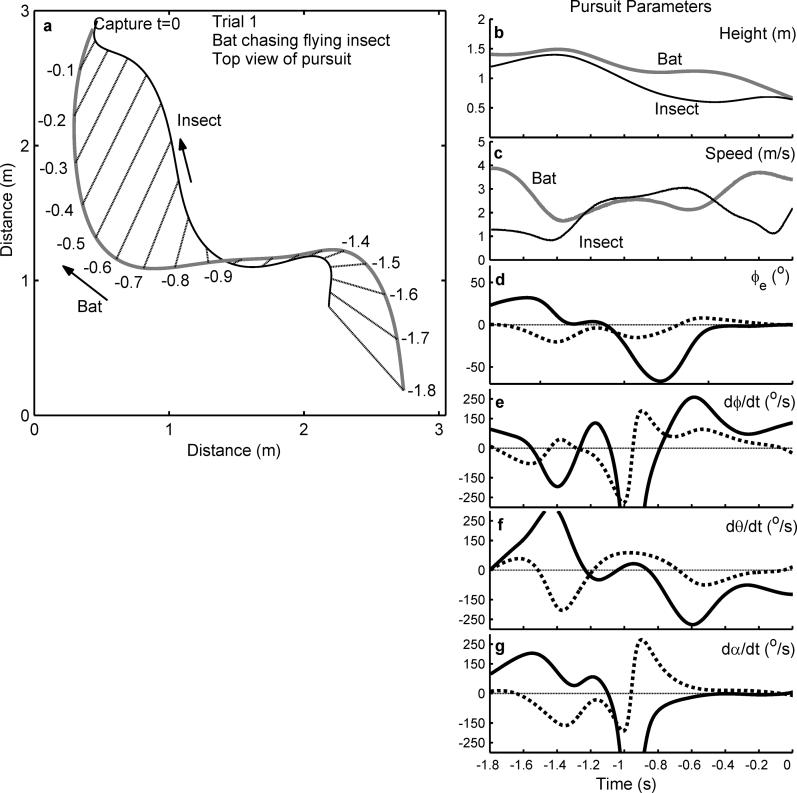
Bat Chasing a Flying Insect (A) Bat (gray line) chases an erratically flying insect (black line) capturing it at time *t* = 0. Bearing lines (black dotted) are drawn from the bat to the target every 100ms. Numbers along the flight path indicate the time in seconds to capture. (B and C) The height (B) and speed (C) of the insect vary continually. (D) The bat maneuvers to drive *φ _e_* → 0 in the horizontal (solid line) and vertical (dotted line). (E) The bearing *φ* is not held constant as *φ _e_* → 0 (solid line, horizontal; dotted line, vertical). (F) The direction of flight ( *θ*) is not held constant (solid line, horizontal; dotted line, vertical). (G) As *φ _e_* → 0, the rate of change of absolute target direction goes to zero (solid line, horizontal; dotted line, vertical). This can also be seen in (A) from the parallel appearance of the dotted lines drawn from the bat to the mantis at 100-ms intervals during the last 700 ms of pursuit (see Figure 3, Videos S1– S4).

During insect capture, the bat maneuvered to maintain an optimum bearing such that *φ _e_* → 0, where *φ _e_* (given by Equation 2) is the difference between the actual target bearing ( *φ*) and the theoretically optimal one ( *φ _opt_,* given by Equation 1). The bat maintained the optimum bearing by keeping the absolute direction to the target a constant ( 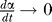). This is illustrated by the example shown in Figure 2. In Figure 2 the bat chased an erratically flying insect. The numbers along the flight path show time in seconds before capture. Solid lines in Figure 2D– 2G are for *horizontal* components of motion, while dotted lines are for *vertical* components. The insect (thin black line) made sudden changes in direction ( Figure 2A, top) and in height ( Figure 2B) while continuously changing speed ( Figure 2C). For the last 500 ms before capture, the bat (thick gray line in Figure 2A– 2C) maneuvered such that *φ _e_* approached zero (see Figure 2D), indicating that it maintained optimum bearing during its pursuit. During this period the bat did not null *dφ/dt* and *dθ/dt* (see Figure 2E and 2F), as would be consistent with a CB strategy. As expected from a CATD strategy *dα/dt* was close to zero during this period ( Figure 2G).

**Figure 3:**
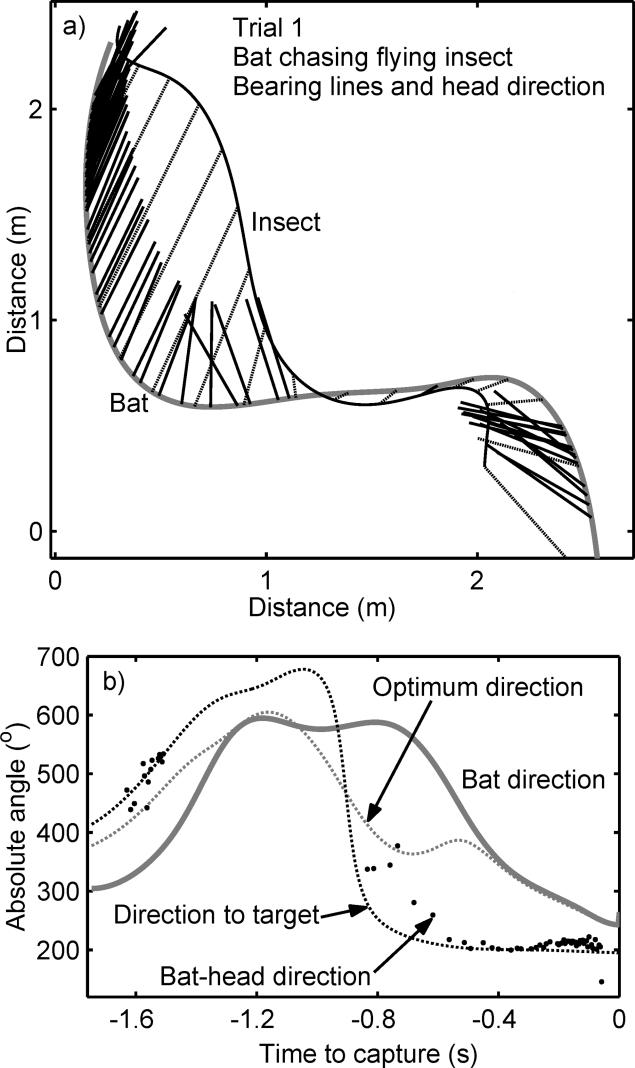
The Bat's Head Is Stabilized in Space during CATD because the Bat Locks Its Head onto the Target and Keeps the Absolute Direction of the Bearing Lines Constant (A) [Similar to Figure 2A] Bat (gray curve) chases an erratically flying insect (black curve) capturing it at time *t* = 0. Bearing lines (black dotted) are drawn from the bat to the target every 100 ms. The head-aim of the bat is computed and drawn (straight black line shooting from bat's flight track) each time it emits a vocalization. (B) The bat's flight direction (thick grey line), the theoretically optimum direction (thin, dotted grey line), the direction to the target (black dotted line), and the bat's head direction (black dots) are shown. Visual inspection of (A) and the computations in graph (B) show that when the bat converges to the CATD strategy (i.e., matches its direction to the optimum direction by maneuvering to optimum bearing), its absolute head direction stabilizes, since it locks onto the target with its head. This can be seen dynamically in Videos S1– S4. We use this observation to suggest, in the discussion, a simple mechanism by which the bat can implement CATD (a functionally predictive strategy) without needing an internal model of target motion.

Figure 3 shows that the bat's head is stabilized in space when it converges to the CATD strategy. The bat head direction is computed from the recorded sonar beam patterns [ 17]. The bat locks its head onto its target during the high repetition rate stage of insect pursuit. This lock is maintained throughout the interception maneuver. This can be seen by inspection in Figure 3A and quantitatively in Figure 3B). From Figure 3B it can be seen that when the bat converges to the optimum direction, it also converges to the CATD strategy (the absolute direction of the target remains constant, appearing as a flat line in Figure 3B) even though its direction of flight keeps changing. Since the bat's head is locked to the target, the absolute direction of the head (black dots) remains constant during this phase of the pursuit. We use this observation to propose, in the discussion section, a biologically plausible mechanism by way of which the bat can achieve the computations required by the CATD strategy.

**Figure 4:**
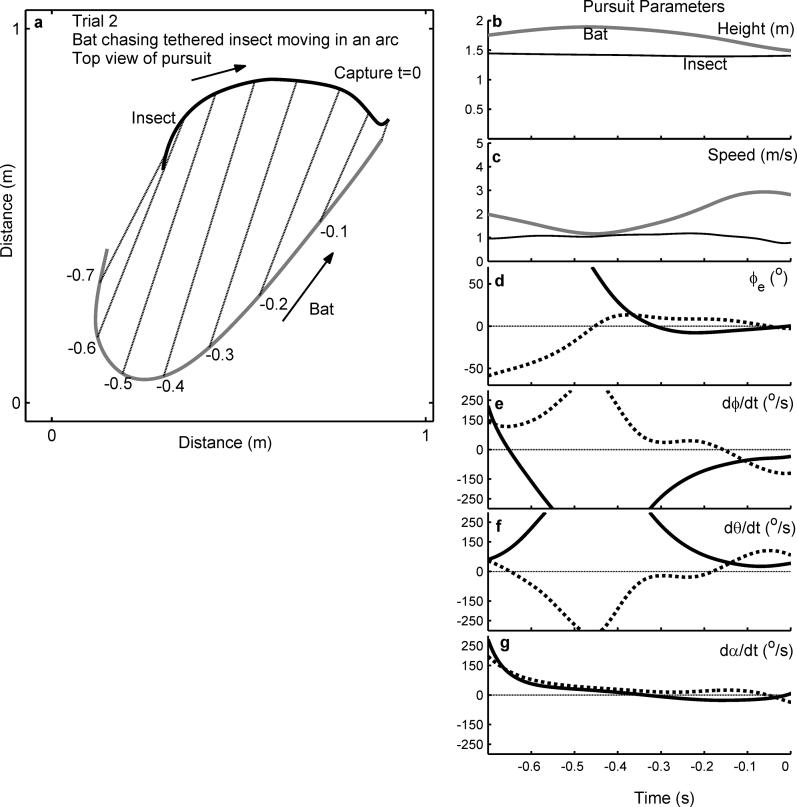
Bat Chasing a Tethered Insect (A) Bat (gray line) chased a tethered insect, moved in an arc (black line), captured it at time *t* = 0. Bearing lines (black dotted) are drawn from the bat to the target every 100ms. Numbers along the flight path indicate the time in seconds to capture. (B and C) The height (B) and speed (C) of the insect were more constant than in Figure 2. (D) The bat maneuvered to drive *φ _e_*→ 0 in the horizontal (solid line) and vertical (dotted line). (E) The bearing ( *φ*) converged to a constant value (solid line, horizontal; dotted line, vertical). (F) The direction of flight ( *θ*) converged to a constant value (solid line, horizontal; dotted line, vertical). (G) The rate of change of absolute target direction converged to zero (solid line, horizontal; dotted line, vertical) before *φ _e_* → 0. This can also be seen in (A) from the parallel appearance of the dotted lines drawn from the bat to the insect at 100-ms intervals.

Figure 4 illustrates a trial in which the bat chased a tethered insect moving in an arc. Solid lines in Figure 4D– 4G are for *horizontal* components of motion, while dotted lines are for *vertical* components. Compared with Figure 2 the tethered target had less variability in height ( Figure 4B) and speed ( Figure 4C). The bat made a U-turn, thereby reducing *φ _e_* to zero ( Figure 4D). In this trial, as *φ _e_* → 0 the rate of change of bearing ( *dφ/dt,* Figure 4E) and flight direction ( *dθ/dt,* Figure 4F) also approached zero, making it difficult to discriminate between the CB and CATD models. In this trial, note that *dα/dt* converges to zero earlier (−600 ms, Figure 4G) than 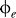 (−300 ms, Figure 4D). In Figure 4A (top) we note that during the first 400 ms the distance from bat to target increased as the bat made a U-turn. *φ _e_* only approaches zero when the bat is able to both match the target's transverse velocity component and simultaneously decrease distance to the target. In the period −0.6 s to −0.3 s, the bat matched the transverse velocity component of the target, but was moving away from it. See Videos S1– S4 to view animations of the bat's pursuit strategy.

**Figure 5:**
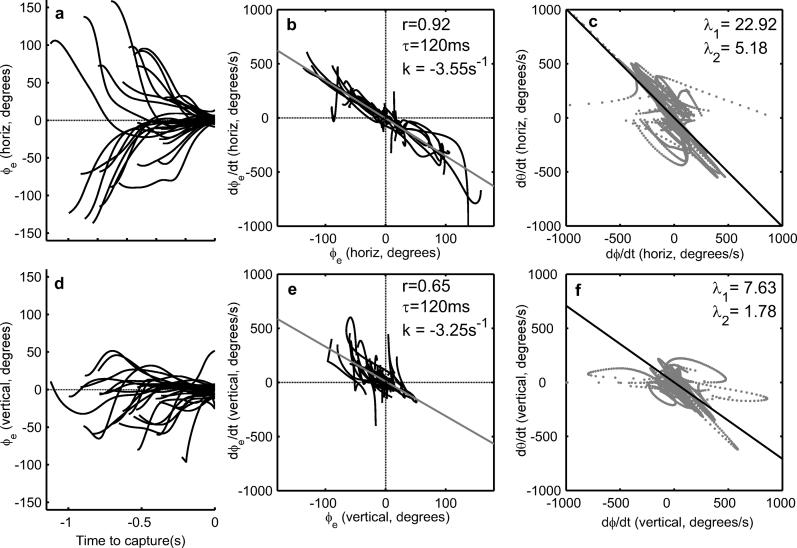
Bats Maneuver to Follow the Optimum Bearing by Keeping *dα/dt* Low *φ _e_* is the deviation of the target bearing from the instantaneous optimum. Data is shown from captures of both free-flying (15 trials) and tethered insects (15 trials) by eight bats. (A) Horizontal component of *φ _e_*. Time of insect capture is *t* = 0. The bat reduces *φ _e_* during pursuit. For clarity each trial is shown from the instant the bat begins to maneuver to reduce *φ _e_*. (B) The pursuit behavior is captured by a delay-differential equation ( Equation 5). The gain in the model is given by *k* = −3.55 s ^−1^, and the delay is given by *τ* = 120 ms. The linear fit has a correlation coefficient r = 0.92. (C) The scatter plot of *dθ/dt* against *dφ/dt* has its principal component ( *λ*_1_) (black line) along *y* = − *x,* indicating that *dφ/dt = −dθ/dt*. (D) Vertical component of *φ _e_* for the same trials and same part of pursuit as in (A). (E) The bat follows a similar law in reducing *φ _e_* in the vertical plane. *k* = −3.2 s ^−1^, τ = 120 ms, r = 0.65. (F) The black line shows the principal component (λ _1_) of *dθ/dt* against *dφ/dt* for vertical components of motion.



To determine if the bat's flight behavior was better described by the CB strategy or the CATD strategy, we analyzed 30 successful insect captures by eight bats. Of these, 15 trials were of the bat capturing free-flying insects, and 15 trials were of the bat capturing tethered insects ( Figure 5). In each case the bat was observed to maneuver to approach the optimum bearing in both horizontal and vertical planes ( Figure 5A and 5D). As can be seen from the plots of *dφ _e_/dt* against *φ _e_* in Figure 5B and 5E, the bat maneuvered to reduce *φ _e_* to zero during pursuit. We were able to model the *φ _e_* data well by a delay-differential equation

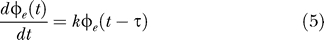



with a negative gain parameter *k* and a delay *τ*. The delay, *τ,* in the model is most likely due to a combination of delays in different parts of the system, including sensorimotor processing time and delay due to the aerodynamics of the bat. It follows from the theory of delay differential equations [ 18] that solutions to Equation 5 are well-posed and unique given any initial condition, 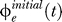, over a time interval of length τ. Moreover, if the gain *k* is negative and the product *kτ* of the gain and time delay is greater than −π/2, each solution is a weighted infinite sum of decaying exponentials, and the decay rate of each term in the sum is given by the roots of the characteristic exponential polynomial *s* − *ke* ^− *τs*^ associated with the delay differential equation ( Equation 5) (see Theorems 4.1 and 13.8 in [ 18], a result due to Hayes [ 19]. This stability constraint on the parameters of the model is met by the estimates of *k* and *τ* in Figures 5B and 5E.

As the bat maneuvers to reduce *φ _e_,* it faces an erratically moving target. We recall from Equation 4 that if the bat follows a CATD strategy, *dα/dt* → 0, resulting in 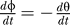. From the experimental data we see that the bat's strategy is not well fit by a CB model (where 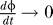) but rather by a CATD model (where 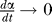, or 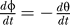). This can be clearly seen in the scatter plot of 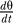 against 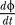 in Figure 5C. The principal component of the data (λ _1_) is along [−1 1] and accounts for 81% of the variance. In the horizontal plane, therefore, the bat keeps 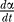 low (→0) at the expense of 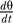 and 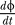. In the vertical plane the principal component (λ _1_) of the scatter of 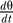 against 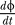 is along [0.82 −0.58], ( Figure 5F, 81% of variance). In the vertical plane, the bat tends to restrict its change in motion ( *dθ/dt*) at the expense of (proportionally) larger changes in bearing angle ( *dφ/dt*) and absolute target direction. One reason for this difference in the bat pursuit strategy along the vertical dimension may be that the bat tends to pounce on the target from above (see Figures 2B and 4B). So in the vertical plane, the bat may not be trying to match up with the target until it gets very close. At a distance the bat may be aiming for a point slightly above the target. The bat's ability to quickly change altitude may also be less than its ability to change direction in the horizontal.

## Discussion

These results show that the bat maneuvers to approach the instantaneous optimal bearing even when the target is moving erratically. In the horizontal plane the bat prefers to keep the absolute direction to the target ( *α*), rather than the target bearing ( *φ*), a constant. Thus the bat, unlike a variety of other animal species, does not use a CB strategy while following its prey. We propose that bats follow a CATD strategy. The bat adjusts its direction of flight and its speed of pursuit so as to maintain the *absolute* direction to the target a constant during pursuit.

When the bat converges to (and maintains) the optimal bearing, the absolute direction to the target does not change. The CATD strategy produces a trajectory which, from the viewpoint of the target, makes the pursuer “appear” stationary against a distant background, and vice-versa. Such trajectories have been observed in the flights of male dragonflies engaged in territorial interactions and have been interpreted as camouflaging behavior on the part of the pursuing male [ 20]. Because motion camouflage is primarily useful for defeating visual detection and the bat reveals its presence and direction with the sonar vocalization, the CATD strategy is unlikely to be employed for camouflage. In ongoing work, we are interested in obtaining a sensorimotor feedback law for implementing the CATD strategy, and a recent paper deriving a feedback law for motion camouflage may serve as a useful guide [ 21]. In the field of missile guidance, the CATD strategy is referred to as parallel navigation. Specific guidance laws to achieve parallel navigation have been developed since the 1940s [ 22, 23]. It appears that a common constraint—the need to intercept unpredictably moving targets as quickly as possible—has driven both engineers and nature to adopt the same strategy.

We propose a simple mechanism that does not require the bat to explicitly compute the quantities in Equation 1 in order to maintain CATD during pursuit. We have shown in an earlier study that the bat locks its head onto a target while chasing it [ 17]. When the bat converges to a CATD strategy, the absolute direction of the bat's head in space is held constant, independent of the orientation of the body and the bat's velocity vector (see Figure 3 for an illustration). The bat could, therefore, maintain CATD by maneuvering to null any changes in head *direction* as sensed by its vestibular system. Because the bat can obtain an accurate estimate of target range through its echolocation system [ 24], it would also sense whether it is approaching the target while holding absolute target direction constant. Alternative mechanisms for following a CATD strategy may involve nulling the apparent motion of the acoustic background, assuming the background sources of noise are distant compared with the target. An interesting possibility is the cross-modal integration of the visual background with the auditory foreground: the bat could follow a CATD strategy by maneuvering such that silhouettes of foliage against the night sky, or the positions of the moon or bright stars (any distant, high contrast object) appear stationary with respect to the acoustically derived position of the target. Some previous modeling studies of bat pursuit behavior have suggested that bats can successfully capture insects using a nonpredictive strategy [ 25, 26], whereas another modeling study has proposed that bats use an internal model of target motion to predictively pursue an insect [ 27]. Our experimental results show that the bat uses a functionally predictive strategy (CATD). The mechanism proposed here, however, allows the bat to implement this functionally predictive strategy without recourse to an internal model of target motion.

From the experiments, we observe that the bat maneuvers to reduce *φ _e_*, the deviation from the optimum direction. We model the experimentally observed data using a delay-differential equation ( Equation 5). In constructing this model we compared linearity between *dφ _e_/dt* and *φ _e_* and *φ _e_* over a range of delays in steps of 4.2 ms (the interval between the video frames) and found that a delay of *τ* = 120 ms produced the best fit (see Figure 5). We hypothesize that this time delay is a combination of physical and biological time delays. Such time delays include *τ _echo_* (the time delay between the emission and reception of the echo), *τ _auditory_* (the time delay incurred in the central nervous system to process sensory input) and *τ _motor_* (the delay due to pre-motor processing and due to the dynamics of the muscular and skeletal system coupled to the aerodynamics). Of these delay components *τ _echo_* is the easiest to estimate: the maximum prey distance is about 2 m, leading to *τ _echo_* ≤ 12 ms under room conditions. It is harder to obtain estimates for the other delays. Neural response latencies to echo stimuli in the bat midbrain can be less than 4 ms and greater than 20 ms [ 28, 29]. A conservative estimate of *τ _auditory_ =* 20 ms, therefore, still leaves a major portion of the delay (about 90 ms or 75%) to be taken up by *τ _motor_*. In this context, bat head movements with a latency of 100 ms are obtained from microstimulation of the bat superior colliculus [ 30] (a midbrain structure implicated in orienting behavior [ 31]). Interestingly, the overall delay of 120 ms that is obtained from our study of bat flight maneuvers is comparable to the latency of 100 ms obtained for human express saccades [ 32].

Since the bat could perform the computations for the CATD strategy by maneuvering to null rotational movements of the head, the bat could link its vestibular system to appropriate flight musculature via a “vestibulo-pursuit reflex,” much like the vestibulocollic reflex. Whereas the traditional vestibulocollic reflex serves to stabilize the head direction when the body posture changes [ 33], the proposed vestibulo-pursuit reflex would serve to stabilize the head direction by appropriately changing the bat's flight direction, enabling the bat to use its brainstem to perform the required CATD computations, using cortical input to modulate the computations over longer timescales.

The bat's strategy is equivalent to following an intercept course to the target at every instant of time, assuming the target will continue moving at its current velocity. The CATD strategy has the important near-optimality property that under a piecewise linear approximation ( Figure 1B) it minimizes time-to-intercept of unpredictably moving targets.

## Materials and Methods

**Figure 6:**
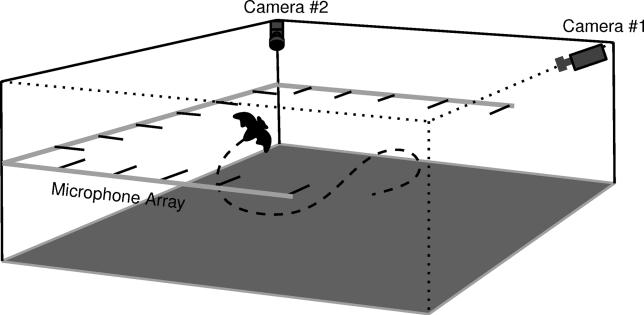
Instrumented Flight Room The bats were trained to fly in a flight room 7.3 m × 6.4 m × 2.5m high. The room walls and ceiling were covered with sound-absorbent foam to reduce reverberations. Illumination was dim red lighting (wavelength >650 nm) to exclude the bat's use of vision. Two digital video cameras operating at 240 frames/s recorded the bats and tethered insects during the experiments.

We used big brown bats *(E. fuscus)* to study sonar guided flight. The sonar pulses produced by these bats are 2–20 ms long, and consist of multiple harmonics with the fundamental sweeping from approximately 60 kHz down to 22 kHz [ 34]. The bats change their pulse production rate (PPR) with behavioral state [ 1]. When searching for prey the PPR is low (2–10 Hz), but as the bat detects and then approaches prey, the PPR rises, terminating in the attack phase (“terminal buzz” [ 1]) where the PPR may be as high as 200 Hz. We trained eight bats to fly in a large (L7.3 m × W6.4 m × H2.5 m) laboratory room ( Figure 6). The room walls and ceiling were lined with sound-absorbent foam to reduce reverberations. The room was illuminated by dim, long wavelength light (>650 nm, light from normal incandescent bulbs filtered through a filter plate: Plexiglas G #2711, Atofina Chemicals, Philadelphia, Pennsylvania, United States) to which the bat is insensitive [ 35]. Images from two high-speed video cameras (Kodak MotionCorder, CCD-based cameras, running at 240 frames/s, synchronized to 1/2 frame accuracy) (Eastman Kodak, Rochester, New York, United States) were used to reconstruct the three-dimensional flight path of the bats and the trajectory of the prey. The reconstruction was done using commercially available motion analysis software (Motus, Peak Performance Technologies, Englewood, Colorado). Simultaneously, a custom built, U-shaped array of 16 microphones recorded horizontal cross-sections of the sonar beam pattern emitted by the bats. Big brown bats emit their echolocation cries through the open mouth, so the axis of the sonar beam is aligned with the axis of the head. These measurements, therefore, allow us to compute the horizontal direction of the bat's head [ 17].

The bats were trained to catch both free flying and tethered insects. Each bat was tested individually as it chased a single prey presented in the room. The free flying insects were a species of praying mantis *(Parasphendale agrionina)*. The mantis was released by hand as the bat was flying around in the room. The mantises had their ears plugged with Vaseline to suppress ultrasound-triggered diving behavior. The mantises made erratic flight maneuvers after release into the room. The tethered insects were inch-long mealworms tethered by a length of monofilament fiber. The tethered insects were concealed in a trapdoor mechanism that was placed at random positions on the ceiling. The bat was allowed to fly in the room for a period of time (10–30 s) after which the prey was released from the trapdoor. The tethered insect was moved in sections of an arc after release by activating a motorized boom attached to the trapdoor assembly. Each bat was tested individually as it chased a single prey presented in the room. A trial consisted of the release of the insect and the first attempt by the bat to capture it.

## Supporting Information

Video S1 Bat Attacking Flying Insect The video has been slowed down by a factor of 10. The bat's position at each frame is depicted by a blue circle and its trajectory is drawn as a blue line. The insect's position is depicted by a black cross and its trajectory is drawn as a black line. The sonar beam patterns depicted in the animations use grayscale to represent sonar beam intensity. Black is the direction of the most intense part of the beam. Shades of gray are linearly scaled to the sound intensity. The computed beam direction for each vocalization is shown as a short black line.
(487 KB WMV)

Video S2 Bat Attacking Flying Insect The video has been slowed down by a factor of 10. The bat's position at each frame is depicted by a blue circle and its trajectory is drawn as a blue line. The insect's position is depicted by a black cross and its trajectory is drawn as a black line. The sonar beam patterns depicted in the animations use grayscale to represent sonar beam intensity. Black is the direction of the most intense part of the beam. Shades of gray are linearly scaled to the sound intensity. Bearing lines from the bat to the insect are drawn at 100-ms intervals.
(502 KB WMV)

Video S3 Bat Attacking Flying Insect The video has been slowed down by a factor of 10. The bat's position at each frame is depicted by a blue circle and its trajectory is drawn as a blue line. The insect's position is depicted by a black cross and its trajectory is drawn as a black line. The sonar beam patterns depicted in the animations use grayscale to represent sonar beam intensity. Black is the direction of the most intense part of the beam. Shades of gray are linearly scaled to the sound intensity. The computed beam direction for each vocalization is shown as a short black line.
(565 KB WMV)

Video S4 Bat Attacking Flying Insect The video has been slowed down by a factor of 10. The bat's position at each frame is depicted by a blue circle and its trajectory is drawn as a blue line. The insect's position is depicted by a black cross and its trajectory is drawn as a black line. The sonar beam patterns depicted in the animations use grayscale to represent sonar beam intensity. Black is the direction of the most intense part of the beam. Shades of gray are linearly scaled to the sound intensity. Bearing lines from the bat to the insect are drawn at 100-ms intervals.
(573 KB WMV)

## Abbreviations

CATD: constant absolute target direction
CB: constant bearing
PPR: pulse production rate
